# Reduced plasma albumin predicts type 2 diabetes and is associated with greater adipose tissue macrophage content and activation

**DOI:** 10.1186/s13098-019-0409-y

**Published:** 2019-02-07

**Authors:** Douglas C. Chang, Xiaoyuan Xu, Anthony W. Ferrante, Jonathan Krakoff

**Affiliations:** 1 Phoenix Epidemiology and Clinical Research Branch, National Institute of Diabetes and Digestive and Kidney Diseases, National Institutes of Health, 4212N. 16th Street, Phoenix, AZ 85016 USA; 2 Department of Medicine, The Naomi Berrie Diabetes Center, Columbia University, New York, NY USA

**Keywords:** Plasma albumin, Type 2 diabetes, Adipose tissue, Inflammation

## Abstract

**Background:**

Plasma albumin is reduced during inflammation. Obesity, a strong risk factor for type 2 diabetes (T2D), is associated with adipose tissue inflammation. However, whether albumin is associated with adipose tissue inflammation and whether it predicts T2D are unclear.

**Methods:**

Adults (predominantly American Indian) from a longitudinal study were included. Macrophage content and gene expression related to recruitment/activation were measured from subcutaneous adipose tissue (n = 51). The relationship between plasma albumin and adiposity (dual-energy X-ray absorptiometry or hydrodensitometry), glucose (oral glucose tolerance test), insulin action (hyperinsulinemic-euglycemic clamp), and insulin secretion (intravenous glucose tolerance test) were evaluated (n = 422). Progression to T2D was evaluated by Cox regression (median follow-up 8.8 years; 102 progressors).

**Results:**

Albumin was associated with macrophage markers including C1QB (r = − 0.30, p = 0.04), CSF1R (r = − 0.30, p = 0.03), and CD11b (r = − 0.36, p = 0.01). Albumin was inversely associated with body fat percentage (r = − 0.14, p = 0.003), fasting plasma glucose (r = − 0.17, p = 0.0003), and 2 h plasma glucose (r = − 0.11, p = 0.03), and was reduced in impaired glucose regulation compared with normal glucose regulation (mean ± SD: 39.4 ± 3.6 g/l and 40.1 ± 3.9 g/l, respectively; p = 0.049). Albumin predicted T2D, even after adjustment for confounders (HR, 0.75; 95% CI 0.58–0.96; p = 0.02; per one SD difference in albumin).

**Conclusions:**

Reduced albumin is associated with an unfavorable metabolic profile, characterized by increased adipose tissue inflammation, adiposity, and glucose, and with an increased risk for T2D.

## Background

Albumin is the most abundant soluble protein in plasma or serum, accounting for about 50% to 60% of total protein [1]. It is synthesized nearly exclusively by the liver and about half is degraded by muscle, liver, and kidneys. Albumin is a versatile protein with a variety of biological roles such as maintaining plasma oncotic pressure, providing > 50% of total antioxidant in normal plasma, and serving as the primary carrier protein for non-esterified fatty acids [1]. In addition, albumin binds and transports various other endogenous molecules (e.g. metal ions, heme, thyroxine, bilirubin) [1]. Although historically linked to malnutrition and protein intake, there is now considerable evidence that low circulating albumin is an indicator of an inflammatory process [1, 2].

Although type 2 diabetes (T2D) was traditionally regarded as a metabolic disease with a defect in insulin action preceding or occurring concurrently with pancreatic beta-cell failure [3]. it is now well-recognized that chronic inflammation is a pathogenic component of T2D and obesity, a strong risk factor for T2D [4]. Diminished insulin action is characterized by chronic inflammation involving infiltration of macrophages into adipose tissue [5]. There is evidence for inflammation as a risk factor for development of T2D specifically in American Indians from a community in the southwestern United States, a group which has a high incidence of T2D and obesity [6, 7]. In this population, adipose tissue markers of macrophage activation were associated with insulin action [8], and elevated peripheral leukocyte count predicted worsening insulin action and the development of T2D [9]. It is unclear whether circulating albumin is associated with either macrophage content or activation within adipose tissue.

There have been a limited number of longitudinal studies that have reported the association between circulating albumin and T2D risk [10–14]. Albumin is a negative acute-phase reactant and lower albumin indicates greater inflammation [15]. Thus, lower circulating albumin might be expected to be associated with increased risk of T2D as a few studies have demonstrated [10, 11]. These results are not consistent as others have shown no association [11, 12], or association between lower albumin and lower risk of T2D [13]. However, to our knowledge, none of these studies reported association between albumin and reference measures of insulin action and secretion or accounted for these as potential confounders.

To further elucidate the role of circulating albumin in T2D, we conducted a series of related investigations in American Indians who were from a community in the Southwestern United States [6, 7] and were without diabetes. First, we hypothesized that adipose tissue inflammation might influence plasma albumin concentrations. Therefore, we evaluated the association between circulating albumin and macrophage content/activation in subcutaneous adipose tissue to characterize albumin as an indicator of obesity-induced inflammation. After finding that lower albumin was associated with several markers of greater adipose tissue inflammation, we hypothesized that lower plasma albumin would be associated with more unfavorable metabolic profile. So, in a larger group from this population, we evaluated the associations between albumin with body composition, glucose and insulin concentrations from the oral glucose tolerance test (OGTT), and reference measures of insulin action and secretion [insulin-stimulated glucose disposal (M) by the hyperinsulinemic-euglycemic clamp (HIEC) and acute insulin response (AIR) by the intravenous glucose tolerance test (IVGTT), respectively]. Third, we hypothesized that individuals with lower albumin are at greater risk of developing T2D.

## Methods

### Study population

Between 1965 and 2007, individuals from an American Indian community in Arizona participated in a longitudinal study of diabetes and its complications [6]. Members at least 5 years old were invited for outpatient research examinations every 2 years which included an OGTT. Some members also participated in a detailed inpatient metabolic study to assess determinants of T2D as previously described [16].

For the current study, we included only those who participated in the detailed inpatient metabolic study, non-pregnant adults (age ≥ 18 years) without T2D at baseline, and participants who were at least 6/8 heritage (defined below) American Indian from the southwest. To evaluate the association between plasma albumin with M and AIR, study visits from which the first HIEC and IVGTT were available were used for the cross-sectional analysis. Diabetes at follow-up and diagnosis date was based on OGTT values at the time of the visit or review of medical records. Classification was according to the 2003 American Diabetes Association criteria [17]. Written informed consent was obtained from all participants. Both studies were approved by the Institutional Review Board of National Institute of Diabetes and Digestive and Kidney Diseases.

### Anthropometrics, body composition, and fixed factors

Body mass index (BMI) (kg/m^2^) was calculated by measured height and weight. Heritage for this particular southwestern American Indian community was determined by self-report and classified into those with full heritage (8/8 heritage) and those with mixed heritage (6/8 or 7/8 heritage) and included as a covariate since heritage was previously associated with greater risk of T2D [18].

Body composition was assessed by hydrodensitometry [19] or by total body dual-energy X-ray absorptiometry (DPX-L; Lunar Radiation, Madison, WI). The absorptiometry measures were converged to comparable hydrodensitometry values, using previously derived equations [20] to calculate percentage of body fat (%fat).

### Oral and intravenous glucose tolerance tests

A 75-g OGTT was performed during baseline and follow-up visits. For the IVGTT, a 25-g intravenous glucose bolus injection over 3 min was administered [21, 22]. AIR was calculated as the mean of the 3, 4, 5 min insulin concentrations minus the fasting concentration [22].

### Hyperinsulinemic-euglycemic clamp

The HIEC occurred after an overnight fast whereupon a primed continuous insulin infusion (40 mU/m^2^/min based by body surface area) was administered for 100 min and 20% dextrose was infused at various rates to maintain a 5.6 mmol/l plasma glucose concentration [23]. M was determined from the last 40 min of the insulin infusions while correcting for the steady-state insulin plasma concentration and endogenous glucose production (EGP). The EGP was measured via primed [3-^3^H]-glucose infusions. M was normalized to estimated metabolic body size (fat-free mass + 17.7 kg) [24].

### Analytic procedures

Plasma glucose concentrations were measured by enzymatic methods. Plasma insulin concentrations were determined by the Herbert modification [25] of the method of Yalow and Berson [26] or automated analyzers (Concept 4, ICN Radiochemicals Inc, Costa Mesa, CA; Access, Beckman Instruments). Values from the later insulin assays were regressed to the original radioimmunoassay. Plasma albumin was measured by the bromocresol purple method (Monarch Chemistry analyzer, Instrumentation Laboratory, Lexington, MA; DADE Behring Dimension RxL Chemistry analyzer, Siemens Medical Solutions, Malvern, PA) in the hospital laboratory.

### Fat biopsy, morphological analysis, and real-time quantitative PCR

Subcutaneous adipose tissue was obtained by percutaneous needle biopsy of the periumbilical fat depot and immediately frozen in liquid nitrogen. Detailed description of the biopsy and measurement of macrophage content were previously described [8]. RNA expression markers evaluated from frozen adipose tissue included CD68, integrin αM (ITGAM)/CD11b, CSF1R, LEP/leptin, ICAM1, CCL-2/MCP-1, plasminogen activator inhibitor type-1 (PAI-1), hypoxia-inducible factor-1α (HIF-1a), vascular endothelial growth factor (VEGF), C1QB, S100A8, tumor necrosis factor (TNF), matrix metalloproteinase-9 (MMP9), and CD11c. Methods involving real-time quantitative PCR were previously described in detail [8].

### Statistical analyses

Statistical analyses were performed using SAS software (SAS Version 9.4 or Enterprise Guide 7.13; SAS Institute, Cary, North Carolina). Non-normally distributed data were log transformed (e.g. M and AIR). Pearson or Spearman correlation analysis was used to quantify the relationships between plasma albumin and variables of interest before and after adjustment for covariates. For comparison of participant characteristics, unpaired t-test, Wilcoxon rank-sum, and Chi squared tests were used where appropriate. To assess albumin as a predictor of incident T2D, a Cox model was used to calculate hazard ratios for development of diabetes, adjusting for baseline age, sex, %fat, heritage, M, AIR, and fasting and 2 h-PG concentrations. All analyses used only baseline measurements because our primary interest was the clinically-relevant predictive value of plasma albumin at a single time-point for subsequent development of T2D. Proportional hazards assumptions were checked by assessment of plots of log[− log(survival)] versus log of survival time and inclusion of a time-dependent interaction term. The follow-up time was truncated to 15 years to satisfy the proportionality assumption. To facilitate comparisons, continuous variables including were standardized (i.e. mean = 0 and SD = 1) and the hazard ratio was reported per SD. Cumulative incidence of diabetes was estimated from the Kaplan–Meier method for participants above and below the median. The association between plasma albumin and residual adipose tissue expression was performed, adjusting for potential confounders (e.g. age, sex, %fat), using linear regression. An alpha level of 0.05 was chosen for analyses.

## Results

Baseline participant characteristics for the cross-sectional, prospective, and adipose tissue analyses are shown in Table 1.

**Table 1:** Baseline characteristics

	Adipose tissue	Cross-sectional analysis	Prospective analysis	
			Non-progressors	Progressors
*n*	51	422	277	102
Male, *n* (%)	33 (65)	243 (58)	171 (62)	43 (42)^c^
Age (years)^a^	31 (23, 39)	27 (23, 32)	26 (22, 31)	28 (24, 32)^d^
Full heritage, *n* (%)	37 (73)	345 (82)	217 (78)	92 (90)^e^
Body weight (kg)^b^	94 (24)	94 (23)	91 (21)	102 (22)^f^
BMI (kg/m^2^)^b^	33 (8)	34 (8)	33 (7)	38 (7)^f^
Body fat (%)^b^	31 (8)	33 (8)	31 (8)	36 (7)^f^
FPG (mmol/l)^b^	4.8 (0.5)	5.0 (0.6)	4.9 (0.6)	5.2 (0.6)^f^
2 h-PG (mmol/l)^b^	6.5 (1.8)	6.9 (1.8)	6.5 (1.6)	7.9 (1.8)^f^
NGR/IGR	33/18	265/157	198/79	41/61
Fasting insulin—OGTT (pmol/L)^a^	155 (89, 272)	153 (76, 271)	139 (69, 236)	236 (132, 382)^f^
M (mg kg_EMBS_^−1^ min^−1^)^a^	2.80 (2.41, 3.86)	2.29 (1.99, 2.87)	2.48 (2.11, 3.19)	2.03 (1.86, 2.64)^f^
AIR (pmol/l)^a^	1347 (860, 2309)	1403 (917, 2132)	1535 (1000, 2215)	1264 (785, 1833)^d^
EGP—basal (mg kg_EMBS_^−1^ min^−1^)^b^	1.95 (0.35)	2.45 (0.40)	1.90 (0.24)	1.91 (0.26)
EGP—insulin (mg kg_EMBS_^−1^ min^−1^)^a^	0.27 (0, 0.60)	0.25 (0, 0.58)	0.19 (0, 0.56)	0.39 (0.15, 0.70)^f^
Albumin (g/l)^b^	39.6 (4.2)	39.8 (4.2)	40.2 (4.3)	38.7 (3.6)^f^

*NGR* normal glucose regulation, *IGR* impaired glucose regulation, *EMBS* estimated metabolic body size = fat-free mass + 17.7, *EGP* endogenous glucose production

^a^Data reported as the median (IQR; 25th to 75th percentile)

^b^Data reported as the mean (SD)

^c^ < 0.001

^d^ < 0.05

^e^ < 0.01

^f^ < 0.0001

### Subcutaneous adipose tissue analysis

**Fig. 1:**
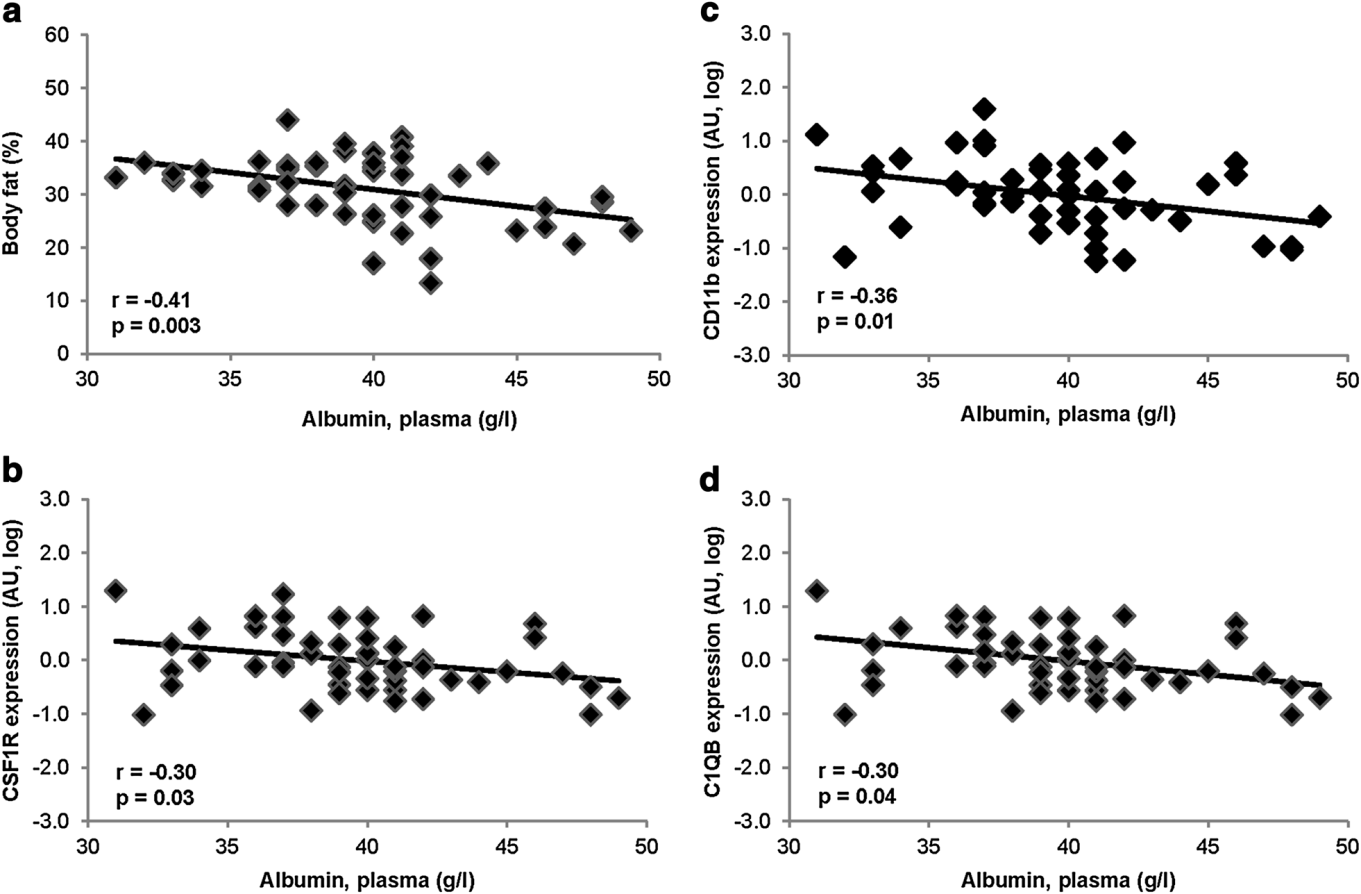
Associations between plasma albumin and **a** body fat percentage (adjusted for age and sex), and **b**–**d** gene expression of inflammatory markers in adipose tissue (adjusted for age, sex, and body fat percentage). AU, mRNA values normalized using mRNA expression of csnk1d

In the group that underwent adipose tissue biopsies, plasma albumin was associated with %fat (r = − 0.41, p = 0.003 l, adjusted for age and sex, Fig. 1a). Plasma albumin was not associated with macrophage content by immunohistochemical analysis. In simple correlations, CD68 and CCL2 were correlated with plasma albumin (r = − 0.37, p = 0.009 and r = − 0.38, p = 0.007, respectively), but not after adjusting for age, sex, and %fat. Plasma albumin was inversely correlated with gene expression markers of adipose tissue macrophage content, CSF1R (r = − 0.30, p = 0.03) and CD11b (r = − 0.36, p = 0.01), adjusted for age, sex, and %fat (Fig. 1b, c, respectively). C1QB, a marker of adipose tissue macrophage activation and a part of the classical complement system mostly expressed in the stromal fraction of adipose tissue [27] and involved in eliciting a macrophage phenotype promoting clearance of apoptotic cells [28], was also associated with plasma albumin (r = − 0.30, p = 0.04; adjusted for age, sex, and %fat; Fig. 1d). These associations indicate that plasma albumin may be reflecting inflammation within adipose tissue.

### Cross-sectional analysis

Plasma albumin declined with increasing age (r = − 0.14, p = 0.004; adjusted for sex and %fat). Men had greater mean plasma albumin compared with women (mean ± SD: 41.1 ± 4.2 g/l and 38.2 ± 4.5 g/l, respectively; p < 0.0001). These sex differences did not persist after adjusting for age and %fat (p > 0.05). Participants of full heritage had lower plasma albumin compared with those less than full heritage (mean ± SD: 39.6 ± 4.1 g/dl and 41.0 ± SD 4.4 g/dl, respectively; p < 0.0001). After adjusting for age, sex, and %fat, this difference was largely attenuated but remained significantly different (mean ± SD: Full 39.9 ± 4.1 g/l; Non-full 39.8 ± 4.7 g/l; p = 0.01).

Plasma albumin was inversely associated with weight (r = − 0.21, p < 0.0001), BMI (r = − 0.33, p < 0.0001), %fat (r = − 0.37, p < 0.0001). Controlling for age, sex, heritage, M, AIR, and FPG and 2 h-PG concentrations attenuated but did not abolish the inverse association between albumin and %fat (r = − 0.14, p = 0.003). Plasma albumin was associated with increasing insulin action (r = 0.10, p = 0.045; adjusted for age and sex), but not significantly correlated after further adjusting for %fat (p > 0.05). Plasma albumin, controlling for age and sex, was not associated with AIR or EGP during the basal and insulin-infusion periods.

Plasma albumin was reduced in participants with impaired glucose regulation (IGR) compared with those with normal glucose regulation (NGR) (mean ± SD: 38.5 ± 4.0 g/l and 40.6 ± 4.2 g/l, respectively; p < 0.0001). The difference remained when adjusted for age, sex, %fat, and heritage (mean ± SD: IGR 39.4 ± 3.6 g/l; NGR 40.1 ± 3.9 g/l; p = 0.049). Lower plasma albumin was associated with higher FPG (r = − 0.29, p < 0.0001). The association between plasma albumin and FPG persisted when adjusting for age, sex, heritage, %fat, M, and AIR (r = − 0.17, p = 0.0003). Reduced albumin was also associated with higher 2h-PG (r = − 0.25, p < 0.0001) and the association remained when controlling for age, sex, %fat, heritage, M, and AIR (r = − 0.11, p = 0.03). Plasma albumin was inversely associated with fasting plasma insulin during the OGTT (r = − 0.10, p = 0.049; adjusted for age and sex) but the association did not persist after further adjustment for %fat.

### Prospective analysis

Of the 422 subjects in the cross-sectional analysis, 379 (90%) had follow-up over a median of 8.8 years [Interquartile Range (IQR), 5.5 to 12.2 years] and 102 developed T2D (Table 1). In the Cox models, continuous variables [plasma albumin, age, %fat, M (log), AIR (log), FPG, and 2h-PG] were standardized and expressed per one SD change in these variables. Reduced albumin predicted progression to T2D in univariate analysis (HR 0.63; 95% CI 0.51–0.78, p < 0.0001) and remained predictive of T2D in different multivariate models using Cox regression (Table 2). In the full model adjusting for age, sex, heritage, %fat, M, AIR, FPG, and 2h-PG, higher albumin was protective for progression to T2D (HR 0.75; 95% CI 0.58–0.96 (Table 2). Cumulative incidence rates of T2D at 10 years were 31% and 16% for the groups below and above the median plasma albumin, respectively, adjusting for the same covariates as the full Cox model. Results were consistent in sensitivity analysis restricting participants to those who were full heritage.

**Table 2:** Cox models for type 2 diabetes in relation to plasma albumin

Model adjustments	HR	(95% CI)	p value
Unadjusted model			
Albumin	0.63	(0.51, 0.78)	< 0.0001
Adjusted model 1			
Albumin	0.71	(0.56, 0.90)	0.005
Adjusted model 2			
Albumin	0.77	(0.60, 0.97)	0.03
Body fat percentage	1.49	(1.12, 1.98)	0.007
Adjusted model 3			
Albumin	0.74	(0.57, 0.95)	0.02
Body fat percentage	1.22	(0.88, 1.69)	0.24
M (log)	0.39	(0.28, 0.55)	< 0.0001
AIR (log)	0.64	(0.53, 0.77)	< 0.0001
Adjusted model 4			
Albumin	0.75	(0.58, 0.96)	0.02
Body fat percentage	1.26	(0.91, 1.77)	0.17
M (log)	0.47	(0.32, 0.68)	< 0.0001
AIR (log)	0.71	(0.53, 0.78)	0.0007
FPG	0.99	(0.77, 1.28)	0.93
2-h PG	1.38	(1.09, 1.75)	0.007

Models 1–4 are also adjusted for age, sex and American Indian heritage

Albumin, body fat percentage, M, AIR, and 2h-PG were standardized to a normal distribution with SD = 1

*AIR* acute insulin response, *FPG* fasting plasma glucose, *2h-PG* 2 h plasma glucose

## Discussion

In this study, complementary analyses delineated the role of plasma albumin in obesity and T2D. Among healthy adult participants, reduced albumin, independent of %fat, was found to be associated with several expression markers of adipose tissue inflammation, indicating that albumin may be reflecting an immunological milieu which may be contributing to risk of T2D. Furthermore, reduced albumin was associated with higher %fat and higher plasma glucose concentration. Moreover, the current study, involving a large sample in a well-defined population with detailed reference measurements of important risk factors for T2D (e.g. %fat, insulin action, and insulin secretion), also demonstrated that lower albumin predicts the development of T2D even when accounting for these confounders.

In cross-sectional studies, those with T2D have lower circulating albumin [29, 30] compared to those without T2D. The current study, demonstrating that lower albumin is associated with higher glucose concentrations and elevates risk of incident T2D, indicates a potential mechanism that is both influencing albumin abundance and contributing to the pathogenesis of T2D. Several possibilities exist for such a mechanism. First, diabetes may result in decreased hepatic albumin synthesis [31, 32]. Second, albumin could be reduced via mechanisms related to formation of glycated albumin [1]. Glycated albumin has aberrant ability to bind various ligands and acts as a precursor to advanced glycation end-products, leading to oxidative stress and inflammation [1]. Given the deleterious consequences of glycated albumin, it is not surprising that mechanisms to clear glycated albumin have evolved. In diabetic rats, renal clearance of albumin is enhanced by glycation [33]. Moreover, there is evidence that glycated albumin (and other glycated proteins) elicits an immunological response that may further reduce albumin. Glycated proteins such as albumin may present new immunological epitopes and hence act as neo-antigens for autoantibody production. Autoantibodies against reactive-oxygen species-modified glycated albumin has been shown to be present in persons with type 1 and T2D [34]. These autoantibodies with their antigen form circulating immune complexes which are predominantly cleared [35]. Albumin has been recently shown to be elevated in these complexes isolated from humans with impaired glucose tolerance and T2D [36], and clearance of these complexes would further lower albumin levels. Glycated albumin was not measured in this study, so its role could not be evaluated. However, there is reason to suspect that relatively increased glycation is occurring among those with low albumin in our study. Higher plasma glucose concentrations and lower albumin were associated, and glycated albumin is an increasingly recognized marker of glycaemia [37]. Another explanation for why higher albumin was protective for T2D may be due to the competition between albumin and other plasma protein for glycation. Due to albumin’s abundance, higher albumin concentration has been shown to protect other proteins such as insulin from being glycated [38], which has been shown to have diminished biological activity as demonstrated by the HIEC procedure [39]. Further studies are needed to elucidate the mechanism behind the association between reduced albumin and increased risk for T2D.

A strength of this study is that the relationships between albumin and adipose tissue markers of inflammation were examined. This finding points to a potential inflammatory source within the body for lower albumin concentrations. Although inflammation within other tissues/organs was not evaluated in this study, other known co-morbidities associated with lower albumin such as infectious or rheumatologic conditions are unlikely given that only healthy individuals were studied. The findings, that reduced albumin is associated with adipose tissue inflammation and predicts T2D, is consistent with chronic low-grade inflammation due to obesity in the development of T2D [15].

The current study found that reduced albumin predicts incident T2D and was consistent with other prospective studies in other populations [10, 11]. However, these results differ from other studies that show no association [11, 12], or association between lower albumin and lower risk of T2D [13]. Differences with prior studies could explain the different results. First, unlike these prior studies, the current study measured adiposity, and insulin action and secretion using reference measures and adjusted for these confounders in the models. Second, the current study was performed in an American Indian population with high rates of obesity and T2D. Though the pathophysiology of the development of diabetes in this American Indian population mirrors those of other populations, it is possible that population differences explain the conflicting results.

Several limitations should be acknowledged. First, since only baseline albumin and risk factors were evaluated, it is possible that accounting for repeated measurements over time could alter the association with risk of T2D. Second, expression of adipose tissue inflammatory genes was available only on a limited number of individuals so the ability of these markers to predict T2D could not be evaluated and compared with albumin. Third, this was an association study, so it cannot be determined whether efforts to maintain the abundance of albumin would be effective in preventing T2D.

## Conclusion

In summary, low albumin was associated with an adverse metabolic profile characterized by increased adiposity, plasma glucose concentrations, and adipose tissue inflammation. Moreover, reduced albumin increased risk for T2D. These findings place albumin within the existing paradigm linking obesity-related inflammation and T2D.

## Abbreviations

2h-PG: 2-h plasma glucose
AIR: acute insulin response
BMI: body mass index
EGP: endogenous glucose production
FPG: fasting plasma glucose
HIEC: hyperinsulinemic-euglycemic clamp
IGR: impaired glucose regulation
IVGTT: intravenous glucose tolerance test
M: insulin-stimulated glucose disposal
NGR: normal glucose regulation
OGTT: oral glucose tolerance test
%fat: percentage of body fat
T2D: type 2 diabetes
